# Calculation of exact Shapley values for explaining support vector machine models using the radial basis function kernel

**DOI:** 10.1038/s41598-023-46930-2

**Published:** 2023-11-10

**Authors:** Andrea Mastropietro, Christian Feldmann, Jürgen Bajorath

**Affiliations:** 1 Department of Computer, Control and Management Engineering “Antonio Ruberti”, Sapienza University of Rome, 00185 Rome, Italy; 2 Department of Life Science Informatics and Data Science, B-IT, LIMES Program Unit Chemical Biology and Medicinal Chemistry, Rheinische Friedrich-Wilhelms-Universität, Friedrich-Hirzebruch-Allee 5/6, 53115 Bonn, Germany

**Keywords:** Drug discovery, Chemistry, Mathematics and computing

## Abstract

Machine learning (ML) algorithms are extensively used in pharmaceutical research. Most ML models have black-box character, thus preventing the interpretation of predictions. However, rationalizing model decisions is of critical importance if predictions should aid in experimental design. Accordingly, in interdisciplinary research, there is growing interest in explaining ML models. Methods devised for this purpose are a part of the explainable artificial intelligence (XAI) spectrum of approaches. In XAI, the Shapley value concept originating from cooperative game theory has become popular for identifying features determining predictions. The Shapley value concept has been adapted as a model-agnostic approach for explaining predictions. Since the computational time required for Shapley value calculations scales exponentially with the number of features used, local approximations such as Shapley additive explanations (SHAP) are usually required in ML. The support vector machine (SVM) algorithm is one of the most popular ML methods in pharmaceutical research and beyond. SVM models are often explained using SHAP. However, there is only limited correlation between SHAP and exact Shapley values, as previously demonstrated for SVM calculations using the Tanimoto kernel, which limits SVM model explanation. Since the Tanimoto kernel is a special kernel function mostly applied for assessing chemical similarity, we have developed the Shapley value-expressed radial basis function (SVERAD), a computationally efficient approach for the calculation of exact Shapley values for SVM models based upon radial basis function kernels that are widely applied in different areas. SVERAD is shown to produce meaningful explanations of SVM predictions.

## Introduction

Machine learning (ML) is a key component of computer-aided drug discovery^1,2^. Fast-growing volumes of chemical and biological discovery data provide a sound basis for the derivation of ML models for practical applications. The data deluge also causes a need for predictive modeling in support of experimental programs. In early-phase drug discovery, many ML applications focus on prediction of candidate compounds with desired biological activity^3–5^. In interdisciplinary research, it is usually required to rationalize predictions for experimental design. However, with the exception of linear regression or simple decision tree models, most ML methods have a black-box character^6^, that is, model decisions cannot be understood by humans, which often limits the impact of ML on experimental programs. Consequently, there is increasing interest in approaches to rationalize ML predictions, which belong to the spectrum of explainable artificial intelligence (XAI) methods^7,8^. For example, among other approaches, methods for model explanation often assess the contributions of input features and identify features that determine predictions^9–18^. While interest in XAI is steadily increasing, the field is far from being mature and relevant approaches are often still in early exploratory stages, which also applies to the chemical sciences and drug discovery^12–15^. Importantly, XAI approaches should not only help domain experts to rationalize predictions, but model explanations should also be accessible to non-expert investigators in interdisciplinary settings^14,15^.

An exemplary generally applicable XAI method is *local interpretable model-agnostic explanations* (LIME)^10^, which determines feature importance for an ML model by performing a local permutation of the input data and deriving a linear approximation. Furthermore, the Shapley value concept^19^ from collaborative game theory has been adapted for quantifying feature importance in ML. The Shapley value formalism was originally designed to determine the contributions of individual players of a team to the performance in a game and divide the gain among players accordingly^19^. Therefore, Shapley values are calculated to quantify the contribution of each player by considering all possible ordered player coalitions. In the XAI adaptation of the Shapley value concept, players correspond to features and the game is the prediction of a test instance. Given the need to enumerate and calculate the marginal contribution of a feature in each possible coalition, the computational requirements of Shapley value calculations scale exponentially with increasing feature numbers. Hence, Shapley value calculations become infeasible for ML models based upon large feature sets. Therefore, corresponding to LIME^10^, an approximation of the Shapley value approach has been introduced, termed *Shapley additive explanations* (SHAP)^20^, representing another model-agnostic approach. For a given non-linear ML model, SHAP derives local linear models in the feature space vicinity of test instances to approximate Shapley values. To this end, Monte Carlo sampling strategies have also been employed^21^ and the SHAP formalism has been extended to cover graph-structured data and graph neural networks^22,23^. Compared to other feature importance methods adapted for the interpretation of quantitative structure–activity relationship (QSAR) models in cheminformatics, a hallmark of the Shapley value/SHAP approach is that it can quantify contributions of features that are present or absent in test instances to their prediction. This is a distinguishing characteristic of the Shapley value/SHAP methodology. Furthermore, algorithms enabling the calculation of exact Shapley values for large feature sets provide a principal advantage compared to local approximation methods. However, another methodology has recently been introduced to adjust the Shapley value formalism for individual data sets^24^, providing an alternative approach compared to the calculation of exact Shapley values in ML.

In XAI, calculation of exact Shapley values has thus far only been accomplished for deriving local explanations of decision tree models^25^ such as random forests (RFs)^26^ and for the support vector machine (SVM) algorithm^27^ in combination with the Tanimoto kernel^28,29^, as recently reported^30^. The decision tree- and SVM-based Shapley value approaches were termed TreeSHAP (or TreeExplainer)^21^ and *Shapley value-expressed Tanimoto similarity* (SVETA)^30^, respectively. Both RF and SVM are for long among the most popular ML methods in pharmaceutical research and other scientific fields, which often rival or exceed the performance of deep neural networks on sets of structured data with well-defined features^15,25^, for example, in molecular property predictions^2,15^. Accordingly, rationalizing SVM black-box predictions is also of considerable interest. Notably, there was only limited correlation between exact Shapley values calculated for the SVM/Tanimoto kernel combination and corresponding SHAP values, indicating that the local approximation might not be suitable for reliable model explanation in this case. Given that the Tanimoto kernel is a special kernel function mostly applied to account for chemical similarity^29^, we devised a methodology for calculating exact Shapley values for SVM models based upon the more generally applied radial basis function (RBF) kernels (including the popular Gaussian kernel). Herein, we report the development and proof-of-concept application of the *Shapley value-expressed radial basis function* (SVERAD) approach yielding exact Shapley values for the SVM/RBF combination in a computationally efficient manner (requiring quadratic computational time with respect to the number of input features). Comparison of SVERAD and SHAP values revealed limited correlation, hence reinforcing the need for calculation of exact Shapley values to explain SVM predictions. As a part of our study, the SVERAD code is made freely available.

## Results

### Scope of the analysis

We first develop the theory and mathematical foundations of SVERAD and then demonstrate the calculation of exact Shapley values using SVERAD based on a model system. In addition, compound activity predictions are carried out using SVM and RF models, and features determining the predictions were identified with SVERAD (SVM), KernelSHAP^20^ (SVM, RF), the general applicable SHAP approximation, and TreeSHAP^25^ (RF). These calculations enabled a direct comparison of SVERAD and SHAP and an additional comparison of corresponding SVM and RF predictions and their explanations. Furthermore, features prioritized for SVM and RF predictions were mapped onto the structures of correctly predicted test compounds to complement numerical analysis and compare chemically intuitive graphical explanations. Finally, XAI analysis is complemented by computational complexity analysis for SVERAD.

### The Shapley value concept

Shapley values represent the weighted average marginal contribution of a feature to a prediction considering all the possible feature coalitions^19,20^. Let $\mathcal{F}$ be the complete set of features and $\mathcal{S}$ a coalition of features (subset of $\mathcal{F}\backslash \{f\}$). The contribution ${\phi }_{f}$ is computed by considering the difference in the value $v$ of the coalition $\mathcal{S}$ with and without the assessed feature $f$, weighted by the inverse multinomial coefficient ${\left(\genfrac{}{}{0pt}{}{\left|\mathcal{F}\right|}{1,\left|\mathcal{S}\right|,\left|\mathcal{F}\right|-\left|\mathcal{S}\right|-1}\right)}^{-1}$, which is calculated as the number of permutations of the coalition ($\left|\mathcal{S}\right|$) multiplied by the number of features not contained in the coalition ($\left|\mathcal{F}\right|-\left|\mathcal{S}\right|-1$) and divided by the number of all possible feature permutations ($\left|\mathcal{F}\right|!$). This must be repeated and summed for all possible subsets $\mathcal{S}$ of the $\mathcal{F}\backslash \{f\}$ features, obtaining the following equation:

$${\phi }_{f}\left(v\right)=\sum_{\mathcal{S}\subseteq \mathcal{F}\backslash \{f\}}\frac{\left|\mathcal{S}\right|!\left(\left|\mathcal{F}\right|-\left|\mathcal{S}\right|-1\right)!}{\left|\mathcal{F}\right|!}\left(v\left(\mathcal{S}\cup f\right)-v\left(\mathcal{S}\right)\right)$$

### Radial basis function kernel

SVM relies on kernel functions for implicitly mapping data distributions into higher-dimensional feature space representations if linear separation of data with different class labels is not possible in a given feature space (“kernel trick”)^31^. For this purpose, alternative kernel functions can be used, depending on the particular applications. Our methodology considers the widely used RBF kernel defined as

$$K\left(\boldsymbol{x},\boldsymbol{x}^{\prime}\right)={e}^{-\frac{{d\left(\boldsymbol{x},\boldsymbol{x}^{\prime}\right)}^{2}}{{2\sigma }^{2}}}$$

where $d\left(\boldsymbol{x},\boldsymbol{x}^{\prime}\right)$ is the Euclidean distance between vectors $\boldsymbol{x}$ and $\boldsymbol{x}^{\prime}$:

$$d\left(\boldsymbol{x},\boldsymbol{x}^{\prime}\right)=\Vert \boldsymbol{x}-\boldsymbol{x}^{\prime}\Vert =\sqrt{\sum_{i}{\left({x}_{i}-{x}_{i}^{\prime}\right)}^{2}}$$

The parameter $\sigma$ is a free parameter used to control the level of nonlinearity of the SVM model that will determine the decision boundary. An alternative definition of the RBF uses the parameter $\gamma =\frac{1}{{2\sigma }^{2}}$, obtaining the equivalent equation

$$K\left(\boldsymbol{x},\boldsymbol{x}^{\prime}\right)={e}^{-\gamma {\Vert \boldsymbol{x}-\boldsymbol{x}^{\prime}\Vert }^{2}}$$

Larger values of $\gamma$ will lead to a more complex decision boundary, while smaller values will render it smoother.

Notably, the RBF function considered is the Gaussian RBF, as it is the most common function employed in kernelized methods and has become a standard in SVM implementations^27^. RBFs are a family of functions with radial symmetry; the Gaussian one is expressed as

$$\varphi (r) = {e}^{-\gamma {r}^{2}}$$

where $r$ is the radial distance, which usually corresponds to the Euclidean distance (as in our case).

In pharmaceutical research, SVM models are mostly derived for molecular property predictions based on chemical structures and therefore employ structural features of compounds as input. Structural features are conventionally encoded in a binary vector format (often termed fingerprints)^32^, that is, a feature can be present or absent in test instance, corresponding to bit settings of 1 or 0, respectively. In the cheminformatics domain, SVMs are currently essentially exclusively employed with binary fingerprint descriptors. Moreover, binary input vectors are also common for other SVM modeling tasks. Therefore, we consider binary encoding of features as a basis for Shapley value calculations (for integer-based representations, adjustments are required). Furthermore, we define $I$ the number of intersecting (common) features between the two feature vectors and $D$ the number of features in the symmetric difference (present in either one vector or the other). ${N}_{i}$ and ${N}_{d}$ will be the number of intersecting and symmetric difference features in a given coalition, respectively. As we show below, the computation of Shapley values using SVERAD only relies on the number of intersecting and symmetric difference features.

### Shapely values for the radial basis function kernel

In order to express feature contributions as Shapley values via the SVERAD formalism, we first need to assess the contribution of features to the Euclidean distance. We notice that features with the same value (intersecting or absent features) do not increase the distance, in fact $\left({x}_{i}-{x}_{i}^{\prime}\right)=0$ if ${x}_{i}={x}_{i}^{\prime}$. Of course, this is true also for non-binary features. Then, features with different values (features with symmetric difference) increase ${d\left(\boldsymbol{x},\boldsymbol{x}^{\prime}\right)}^{2}$ by ${\Delta }_{d}={\left(0-1\right)}^{2}={\left(1-0\right)}^{2}=1$. This leads to having $d\left(\boldsymbol{x},\boldsymbol{x}^{\prime}\right)=\sqrt{{N}_{d}}$ and ${d\left(\boldsymbol{x},\boldsymbol{x}^{\prime}\right)}^{2}={N}_{d}$, indicating that only features with symmetric difference determine the distance (and kernel) value:

$${e}^{-\frac{d(\boldsymbol{x},\boldsymbol{x}^{\prime}{)}^{2}}{2{\sigma }^{2}} }= {e}^{-\frac{{N}_{d}}{2{\sigma }^{2}}}$$

This allows for a fast calculation of the kernel value.

Now, we consider a coalition of features $$\mathcal{S}$$ whose value $$v\left(\mathcal{S}\right)$$ is the RBF kernel value. If $$\mathcal{S}$$ contains intersecting features only ($${N}_{d}=0)$$ we have $$v\left(\mathcal{S}\right)={e}^{-\frac{{N}_{d}}{2{\sigma }^{2}} }={e}^{0}=1$$. This is true for any value of $$I$$ (size of the intersection). Differently, for a coalition with features with symmetric difference only (or with a mixture of intersecting and symmetric difference features), the value $${v\left(\mathcal{S}\right)=e}^{-\frac{{N}_{d}}{2{\sigma }^{2}}}$$ must be calculated given $${N}_{d}$$ and $$\sigma$$ (or $$\gamma$$) , as aforementioned. Finally, for the empty coalition $$\mathcal{S}=\varnothing$$, we set $$v\left(\mathcal{S}\right)=0$$, conforming to the Shapley value formalism for the empty set^16,17^.

To obtain the Shapley values for the RBF kernel, we need to compute the change in the kernel value when a feature from the intersection ${f}_{+}$, or a feature from the symmetric difference ${f}_{-}$, are added to the coalition $\mathcal{S}$ with ${N}_{i}$ intersecting features and ${N}_{d}$ features with symmetric difference. For ${f}_{+}$ we have

$${\Delta v}_{{f}_{+}}\left({N}_{i},{N}_{d}\right)={e}^{-\frac{{N}_{d}}{2{\sigma }^{2}} }-{ e}^{-\frac{{N}_{d}}{2{\sigma }^{2}} }=0$$

Adding a feature from the intersection does not change the distance and thus not the kernel value. This is always true except if ${f}_{+}$ is added to the empty coalition ($v(\varnothing )=0)$. In this case, the kernel value when adding the features becomes 1 (${N}_{d}=0$) and so

$${\Delta v}_{{f}_{+}}\left(\mathrm{0,0}\right)=1$$

Then, for a symmetric difference feature ${f}_{-}$ we have

$${\Delta v}_{{f}_{-}}\left({N}_{i},{N}_{d}\right)={e}^{-\frac{{N}_{d}+1}{2{\sigma }^{2}} }-{ e}^{-\frac{{N}_{d}}{2{\sigma }^{2}}}$$

When adding a feature with symmetric difference, the squared Euclidean distance increases by 1 (as shown). The change in the kernel value must be calculated consequently. When the subtracted term represents the empty coalition, its value is set to 0.

Once we have computed the value change, we need to calculate the number of occurrences for each possible coalition with ${N}_{i}$ intersecting features and ${N}_{d}$ features with symmetric difference. For ${f}_{+}$ we thus consider all possible combinations of ${N}_{i}$ elements in a set of $I-1$ elements (the assessed feature is not a part of the coalition) and ${N}_{d}$ elements in a set of $D$ elements:

$${C}_{{f}_{+}}\left({N}_{i},{N}_{d}\right)=\left(\genfrac{}{}{0pt}{}{I-1}{{N}_{i}}\right)\left(\genfrac{}{}{0pt}{}{D}{{N}_{d}}\right)$$

Likely, for ${f}_{-}$ we consider all possible combinations of ${N}_{i}$ elements in a set of $I$ elements and ${N}_{d}$ elements in a set of $D-1$:

$${C}_{{f}_{-}}\left({N}_{i},{N}_{d}\right)=\left(\genfrac{}{}{0pt}{}{I}{{N}_{i}}\right)\left(\genfrac{}{}{0pt}{}{D-1}{{N}_{d}}\right)$$

Once we have all the elements, we can compute the Shapley values as the sum of the products of ${\Delta v}_{f}$, ${C}_{f}$ and the inverse multinomial coefficient.

For an intersecting feature, the Shapley value (${\phi }_{f}$) for the RBF kernel will be computed as

$${\phi }_{{f}_{+}}=\sum_{{N}_{i}=0}^{I-1}\sum_{{N}_{d}=0}^{D}{\Delta v}_{{f}_{+}}\left({N}_{i},{N}_{d}\right)\cdot {C}_{{f}_{+}}\left({N}_{i},{N}_{d}\right)\cdot {\left(\genfrac{}{}{0pt}{}{I+D}{1,{N}_{i}+{N}_{d,}, I+D - {N}_{i} - {N}_{d} - 1}\right)}^{-1}$$

As previously shown, ${\Delta v}_{{f}_{+}}\left({N}_{i},{N}_{d}\right)$ is always 0, except if ${f}_{+}$ is added to the empty coalition (${N}_{i}=0$ and ${N}_{d}=0$). In this case, the kernel value changes from 0 to 1, thus ${\Delta v}_{{f}_{+}}\left(\mathrm{0,0}\right)=1$. So, we can easily compute the Shapley value considering only the addition to the empty coalition:

$$\begin{aligned} \phi _{{f_{ + } }} & = \Delta v_{{f_{ + } }} \left( {0,0} \right) \cdot C_{{f_{ + } }} \left( {0,0} \right) \cdot \left( {\begin{array}{*{20}c} {I + D} \\ {1,N_{i} + N_{d} ,~I + D~ - ~N_{i} - ~N_{d} ~ - ~1} \\ \end{array} } \right)^{{ - 1}} \\ & = 1 \cdot 1 \cdot \frac{{\left( {N_{i} + N_{d} } \right)!\left( {I + D - N_{i} - N_{d} - 1} \right)!}}{{\left( {I + D} \right)!}} \\ & = \frac{{\left( {I + D - 1} \right)!}}{{\left( {I + D} \right)!}} \\ \end{aligned}$$

Analogously, for a symmetric difference feature, we obtain

$$\begin{aligned} \phi _{{f_{ - } }} & = \mathop \sum \limits_{{N_{i} = 0}}^{I} \mathop \sum \limits_{{N_{d} = 0}}^{{D - 1}} \Delta v_{{f_{ - } }} \left( {N_{i} ,N_{d} } \right) \cdot C_{{f_{ - } }} \left( {N_{i} ,N_{d} } \right) \cdot \left( {\begin{array}{*{20}c} {I + D} \\ {1,N_{i} + N_{{d,}} ,~I + D~ - ~N_{i} - ~N_{d} ~ - ~1} \\ \end{array} } \right)^{{ - 1}} \\ & = \mathop \sum \limits_{{N_{i} = 0}}^{I} \mathop \sum \limits_{{N_{d} = 0}}^{{D - 1}} \left( {e^{{ - \frac{{N_{d} + 1}}{{2\sigma ^{2} }}~}} ~ - ~e^{{ - \frac{{N_{d} }}{{2\sigma ^{2} }}~}} } \right) \cdot \left( {\begin{array}{*{20}c} I \\ {N_{i} } \\ \end{array} } \right)\left( {\begin{array}{*{20}c} {D - 1} \\ {N_{d} } \\ \end{array} } \right) \cdot \frac{{\left( {N_{i} + N_{d} } \right)!\left( {I + D - N_{i} - N_{d} - 1} \right)!}}{{\left( {I + D} \right)!}} \\ & = \mathop \sum \limits_{{N_{i} = 0}}^{I} \mathop \sum \limits_{{N_{d} = 0}}^{{D - 1}} \left( {e^{{ - \frac{{N_{d} + 1}}{{2\sigma ^{2} }}~}} ~ - ~e^{{ - \frac{{N_{d} }}{{2\sigma ^{2} }}~}} } \right) \cdot \frac{{I!}}{{\left( {I - N_{i} } \right)!N_{i} !}} \cdot \frac{{\left( {D - 1} \right)!}}{{\left( {D - N_{d} - 1} \right)!N_{d} !}} \cdot \frac{{\left( {N_{i} + N_{d} } \right)!\left( {I + D - N_{i} - N_{d} - 1} \right)!}}{{\left( {I + D} \right)!}} \\ \end{aligned}$$

The computation can be further simplified by aggregating common factors. The possible coalitions to which ${f}_{-}$ can be added include the empty coalition (${N}_{i}={N}_{d}=0$), coalitions with intersecting features only (${N}_{d}=0$ and $v\left(\mathcal{S}\right)=1$), and coalitions with intersecting and symmetric difference features, or with symmetric difference features only (${N}_{i}\in \left[0,I\right]$ and ${N}_{d}\in \left[1,D-1\right]$). We thus obtain

$$\begin{aligned} \phi _{{f_{ - } }} ~ = ~ & e^{{ - \frac{1}{{2\sigma ^{2} }}~}} \cdot \frac{{\left( {I + D - 1} \right)!}}{{\left( {I + D} \right)!}} + \left( {~e^{{ - \frac{1}{{2\sigma ^{2} }}~}} - 1} \right) \cdot \mathop \sum \limits_{{N_{i} = 1}}^{I} \left( {\begin{array}{*{20}c} I \\ {N_{i} } \\ \end{array} } \right) \cdot \frac{{N_{i} !\left( {I + D - N_{i} - 1} \right)!}}{{\left( {I + D} \right)!}} \\ & + \mathop \sum \limits_{{N_{i} = 0}}^{I} \mathop \sum \limits_{{N_{d} = 1}}^{{D - 1}} \left( {e^{{ - \frac{{N_{d} + 1}}{{2\sigma ^{2} }}~}} ~ - ~e^{{ - \frac{{N_{d} }}{{2\sigma ^{2} }}~}} } \right) \cdot \left( {\begin{array}{*{20}c} I \\ {N_{i} } \\ \end{array} } \right)\left( {\begin{array}{*{20}c} {D - 1} \\ {N_{d} } \\ \end{array} } \right) \cdot \frac{{\left( {N_{i} + N_{d} } \right)!\left( {I + D - N_{i} - N_{d} - 1} \right)!}}{{\left( {I + D} \right)!}} \\ \end{aligned}$$

### Proof-of-concept

To establish initial proof-of-concept for the approach, we calculate Shapley values for the RBF kernel and two exemplary small model vectors $\boldsymbol{x}$ and $\boldsymbol{y}$ using SVERAD:

$$\boldsymbol{x}=\left[1 0 0 1 0\right], \boldsymbol{y}=\left[1 0 1 1 1\right]$$

Notably, these vectors are only used to illustrate the SVERAD calculations and do not represent (high-dimensional) molecular fingerprints. The model vectors share two features (set to 1, intersecting features), so $I=2$, have a unique feature each (set to 0 and 1, respectively, symmetric difference), so $D=2$, and lack a feature (set to 0). For the exemplary calculation, we set $\sigma =1$. Tables 1 and 2 show the steps needed to compute the Shapley values for intersecting and symmetric difference features, respectively.

**Table 1:** Calculation of the Shapley value for an intersecting feature.

${N}_{i}$	${N}_{d}$	$v\left(\mathcal{S}\right)$	$v\left(\mathcal{S} \cup {f}_{+}\right)$	${\Delta v}_{f}$	# coalitions	Inverse multinomial coefficient	${\Delta v}_{f}$⋅ # coalitions ⋅ inv. mult. coeff
0	0	0	1	1	1 ⋅ 1 = 1	¼ = 0.25	0.25
0	1	${e}^{-1/2}$	${e}^{-1/2}$	0	1 ⋅ 2 = 2	1/12 = 0.083	0
0	2	${e}^{-1}$	${e}^{-1}$	0	1 ⋅ 1 = 1	1/12 = 0.083	0
1	0	1	1	0	1 ⋅ 1 = 1	1/12 = 0.083	0
1	1	${e}^{-1/2}$	${e}^{-1/2}$	0	1 ⋅ 2 = 2	1/12 = 0.083	0
1	2	${e}^{-1}$	${e}^{-1}$	0	1 ⋅ 1 = 1	¼ = 0.25	0

**Table 2:** Calculation of the Shapley value for a symmetric difference feature.

${N}_{i}$	${N}_{d}$	$v\left(\mathcal{S}\right)$	$v\left(\mathcal{S} \cup {f}_{-}\right)$	${\Delta v}_{f}$	# coalitions	Inverse multinomial coefficient	${\Delta v}_{f}$⋅ # coalitions ⋅ inv. mult. coeff
0	0	0	${e}^{-1/2}$	${e}^{-1/2}$	1 ⋅ 1 = 1	¼ = 0.25	0.1516
0	1	${e}^{-1/2}$	${e}^{-1}$	${e}^{-1}-{ e}^{-1/2}$	1 ⋅ 1 = 1	1/12 = 0.083	− 0.0199
1	0	1	${e}^{-1/2}$	${e}^{-1/2}-1$	2 ⋅ 1 = 2	1/12 = 0.083	− 0.0656
1	1	${e}^{-1/2}$	${e}^{-1}$	${e}^{-1}{ -e}^{-1/2}$	2 ⋅ 1 = 2	1/12 = 0.083	− 0.0398
2	0	1	${e}^{-1/2}$	${e}^{-1/2}-1$	1 ⋅ 1 = 1	1/12 = 0.083	− 0.0328
2	1	${e}^{-1/2}$	${e}^{-1}$	${e}^{-1}{ -e}^{-1/2}$	1 ⋅ 1 = 1	¼ = 0.25	− 0.0597

As discussed, calculation of the kernel value only depends on the number of features with symmetric difference, resulting in equation

$$K\left(\boldsymbol{x},\boldsymbol{y}\right)={ e}^{-\frac{{N}_{d}}{2} }={ e}^{-1 }=0.368$$

The sum of the Shapley values for all features yields the kernel value. To compute the Shapley value for a feature in the intersection and a feature with symmetric difference, ${\Delta v}_{f}$ is multiplied by the number of coalitions and the inverse multinomial coefficient and the sum over all possible coalitions is calculated. Given that any feature from the same set (intersection or symmetric difference) makes the same contribution to the kernel value, we need to multiply the Shapley value obtained for one representative feature of each set by $I$ and $D$ to obtain the total contribution of the intersecting and symmetric difference features, respectively. In our example, the Shapley value for an intersecting feature is 0.25 and for a feature with symmetric difference it is -0.066. The set of intersecting features ($I=2$) yields a sum of Shapley values of 0.5 while symmetric difference features ($D=2$) contribute to the kernel value for -0.132. The sum of these values is 0.368, which is exactly the kernel value.

As an additional proof-of-concept calculation, we consider 20 random binary vectors with a small number of features ($\left|F\right|=15$), so that Shapley values can be computed explicitly by enumerating all possible coalitions^19^. SVERAD yields the same Shapley values as produced by the exhaustive computation, thus demonstrating the validity of the method. This is also evident in Table 3, which shows a comparison of SVERAD Shapley values with the SHAP approximation (for the calculations, we set $\gamma =\frac{1}{{2\sigma }^{2}}=1$).

**Table 3:** Comparison of exact Shapley values, SVERAD, and SHAP values using Pearson's *r* correlation coefficient with standard deviations.

	Exact Shapley values	SVERAD	SHAP
Exact Shapley values	1.0 ± 0.0	1.0 ± 0.0	0.72 ± 0.43
SVERAD	1.0 ± 0.0	1.0 ± 0.0	0.72 ± 0.43
SHAP	0.72 ± 0.43	0.72 ± 0.43	1.0 ± 0.0

The correlation coefficient of 1 for SVERAD Shapley values and exact Shapley values confirms that both calculations return the same values (the associated error resulting from the imprecision in the representation of very small numbers is smaller than ${10}^{-10}$). This differs from exact Shapley values vs. SHAP, for which a Fisher-transformed correlation coefficient of 0.72 ± 0.43 is obtained, reflecting the underlying local approximation of SHAP values.

It also follows that the predictive performance of original SVM models is not affected through the Shapley value modification because it exactly accounts for the RBF kernel value, as demonstrated above, and the SVM computational classification criteria do not change.

### Shapley values for support vector machine predictions

In an SVM model, the distance of a vector $\boldsymbol{x}$ from the separating hyperplane is defined by the support vectors $\boldsymbol{{V}_{n}}$ and is given by

$$dist\left(\boldsymbol{x}\right)=b+{\sum }_{n=0}^{{N}_{v}-1}{y}_{n}{w}_{n}K\left(\boldsymbol{x},\boldsymbol{{V}_{n}}\right)$$

where ${N}_{v}$ is the number of support vectors, ${y}_{n}$ (-1 or 1) is the class label of the support vector $\boldsymbol{{V}_{n}}$, ${w}_{n}$ is the weight by which the class label is scaled and $K\left(\boldsymbol{x},\boldsymbol{{V}_{n}}\right)$ is the kernel value comparing the support vector and the predicted instance $\boldsymbol{x}$. Finally, $b$ is a bias value.

To compute the Shapley value for the distance for each feature $f$, we first substitute the kernel value with the sum of Shapley values of the features for the RBF kernel between vector $\boldsymbol{x}$ and support vector $\boldsymbol{{V}_{n}}$ (${\phi }_{f,n}$) and scale the sum by the label and the weight. Then, we consider the bias as an additional feature whose value $b$ is its Shapley value:

$$\begin{aligned} dist\left( \boldsymbol{x} \right) &= b + \mathop \sum \limits_{n = 0}^{Nv - 1} y_{n} w_{n} K\left( {\boldsymbol{x},\boldsymbol{V_{n}} } \right) = b + \mathop \sum \limits_{n = 0}^{Nv - 1} y_{n} w_{n} \mathop \sum \limits_{f = 0}^{\left| F \right| - 1} \phi_{f,n} \\ & = b + \mathop \sum \limits_{f = 0}^{\left| F \right| - 1} \mathop \sum \limits_{n = 0}^{Nv - 1} y_{n} w_{n} \phi_{f,n} = \phi_{b} + \mathop \sum \limits_{f = 0}^{\left| F \right| - 1} \phi_{f} \\ \end{aligned}$$

Finally, given the additivity property of Shapley values, the Shapley value for a feature $f$ is obtained by summing up the Shapley values of $f$ for the RBF kernel values comparing vector $\boldsymbol{x}$ with all the support vectors:

$${\phi }_{f}={\sum }_{n=0}^{Nv-1}{y}_{n}{w}_{n}{\phi }_{f,n},$$

which gives the contribution of feature $f$ with respect to the distance from the separating hyperplane.

#### Expressing feature contributions as log odds values

The distance from the hyperplane can be transformed into probability estimates using Platt scaling^33^:

$$p\left(\boldsymbol{x}\right)=\frac{1}{1+{e}^{A\cdot dist\left(\boldsymbol{x}\right)+B}}$$

Given that Shapley values for probabilities cannot be calculated from Shapley values for the distance from the hyperplane, we need to compute the logits (log odds):

$$logit\left(p\left(\boldsymbol{x}\right)\right)=log\left(\frac{p\left(\boldsymbol{\boldsymbol{x}}\right)}{1-p\left(\boldsymbol{x}\right)}\right)=\cdots =log\left(\frac{1}{{e}^{A\cdot dist\left(\boldsymbol{x}\right)+B}}\right)=-A\cdot dist\left(\boldsymbol{x}\right)-B$$

We can express $dist\left(\boldsymbol{x}\right)$ as the sum of the Shapley values for the distance:

$$logit\left(p\left(\boldsymbol{x}\right)\right)=-A\cdot \left({\phi }_{b}+{\sum }_{f=0}^{\left|F\right|-1}{\phi }_{f}\right)-B=-\left(A\cdot {\phi }_{b}+B\right)-{\sum }_{f=0}^{\left|F\right|-1}A\cdot {\phi }_{f}$$

Logits are a linear transformation of the distance. Hence, the Shapley values for the logits are obtained as a linear transformation of the Shapley values for the distance (scaling by $-A$). Moreover, by scaling ${\phi }_{b}$ by $-A$ and offsetting it by $-B$ the Shapley value for the additional feature is obtained, analogously to the Shapley value for the distance bias $b$, previously calculated. The term $-\left(A\cdot {\phi }_{b}+B\right)$ is regarded as an expected value since it does not depend on other features. The sum of the Shapley values $-{\sum }_{f=0}^{\left|F\right|-1}A\cdot {\phi }_{f}$ represents the difference between the actual value and the expected value, conforming to the Shapley value formalism^19,20^.

#### Feature contributions to the radial basis function kernel

For a direct comparison, SVERAD and SHAP values were calculated for 50 randomly selected adenosine receptor A3 ligands (A3 ligands) that we also used for compound activity predictions (see Methods). Compounds were represented using topological structural features, that is, systematically calculated pathways originating from atoms with a constant bond radius (see Methods). The RBF kernel was computed for all possible compound pairs and for each pair, exact Shapley values calculated using SVERAD were compared to corresponding SHAP values from KernelSHAP. For a value $\gamma =\frac{1}{{2\sigma }^{2}}=0.005$, a mean Pearson’s *r* correlation coefficient after Fisher transformation of 0.36 ± 0.18 was obtained. The low correlation indicated that the SHAP approximation was limited in its ability to explain RBF-based similarities and that calculation of exact Shapley values was preferred.

#### Rationalizing compound activity predictions

To apply the SVERAD approach to pharmaceutically relevant predictions and compare model explanations for different Shapley value/SHAP calculation variants, we derived SVM and RF classification models based to distinguish A3 ligands from other randomly selected compounds (see Methods). The SVM and RF classifiers achieved comparably high prediction accuracy of 93% and 92%, respectively. We then analyzed these predictions in detail.

#### Feature contributions to classification models

**Figure 1:**
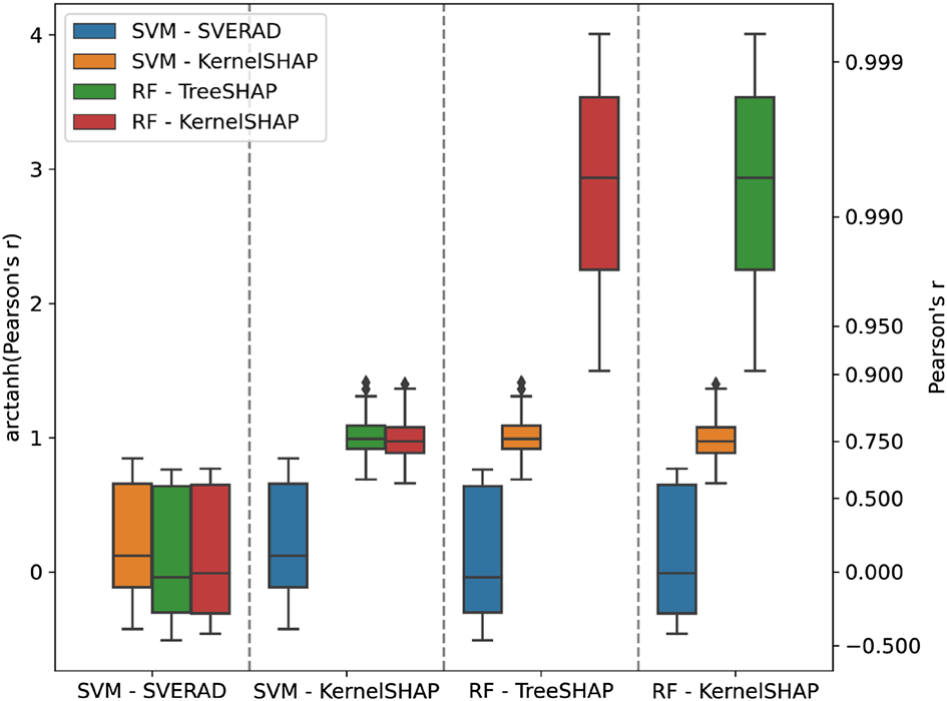
Distributions of Pearson’s *r* correlation coefficient. Box plots represent the distributions of correlation coefficients for feature contributions from different models and explanation strategies.

For SVM predictions, Shapley values and SHAP values were calculated with SVERAD and KernelSHAP and for RF predictions with TreeExplainer and KernelSHAP, respectively. In Table 4, median Pearson’s *r* correlation coefficients are reported for feature contributions and all combinations of classification models and corresponding Shapley value/SHAP calculation methods. In addition, Fig. 1 shows the corresponding distributions of correlation coefficients.

**Table 4:** Median Pearson’s *r* correlation coefficient between feature contributions from different models and explanation strategies.

	SVM – SVERAD	SVM – KernelSHAP	RF – TreeSHAP	RF – KernelSHAP
SVM – SVERAD	1.000	0.120	− 0.040	− 0.010
SVM – KernelSHAP	0.120	1.000	0.758	0.750
RF – TreeSHAP	− 0.040	0.758	1.000	0.994
RF – KernelSHAP	− 0.010	0.750	0.994	1.000

There was only very low correlation between SVERAD Shapley values and SHAP values (0.120), which reflected the apparent limited ability of SHAP calculations to approximate Shapley values for SVM. Notably, the correlation for the SVM/RBF combination was much lower than previously determined for the SVM/Tanimoto kernel combination (0.682)^30^, which reinforced the need for calculating exact Shapley values if the widely applied RBF kernel is used. By contrast, for RF, there was nearly perfect correlation between KernelSHAP and TreeExplainer (0.994), which uses exact Shapley/SHAP values for deriving local explanations. When comparing exact Shapley/SHAP values from SVERAD and TreeExplainer for corresponding predictions, essentially no correlation was observed (-0.040), indicating that different features were determining SVM and RF predictions in the presence of comparably high prediction accuracy. However, in this case, potential correlation was also principally limited because the calculations were based on different metrics (log odds scores for SVM vs. class probabilities for RF). Furthermore, SHAP values for SVM and RF displayed relatively high correlation (0.758). Taken together, the results indicated that SVERAD values were more accurate for SVM using the RBF kernel than the SHAP approximation, whereas TreeExplainer and KernelSHAP values were strongly correlated for RF.

#### Model explanations and feature mapping

**Figure 2:**
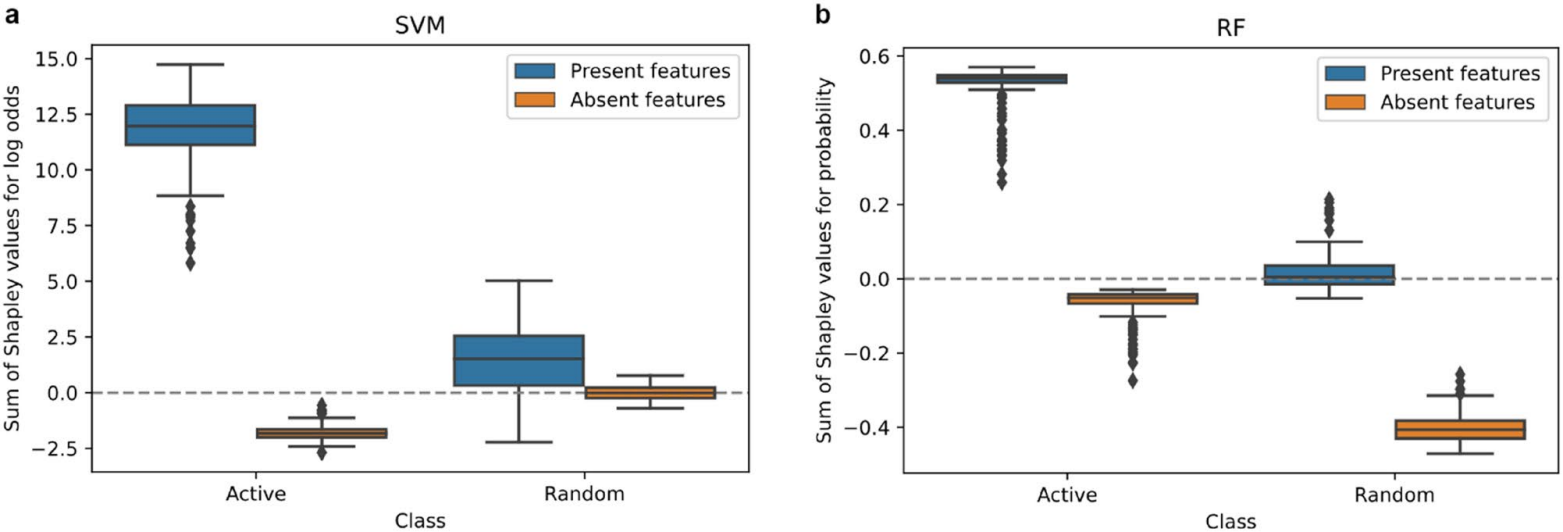
Distribution of feature contributions. Box plots show the distributions of cumulative Shapley values of features present or absent in correctly predicted test instances for SVERAD/SVM (**a**) and TreeExplainer/RF (**b**).

For the SVM and RF predictions, SVERAD and TreeExplainer values were calculated, respectively, and separately analyzed for features that there were present or absent in correctly predicted test compounds. Figure 2 shows the distribution of cumulative Shapley values for these features in test compounds for log odds scores from SVERAD and probabilities from TreeExplainer.

The analysis explained model decisions and revealed different prediction characteristics for SVM and RF. For SVM, features present in active compounds made strong positive contributions to correct predictions, whereas absent features made only minor contributions to incorrect predictions. For random compounds, present features made small contributions to incorrect predictions (of activity) while absent features made essentially no contributions (with cumulative Shapley values close to zero). Hence, correct predictions of inactive compounds can only be rationalized taking the expected values into account, as discussed below. For RF, features present in active compounds determined their correct predictions, while the absence of these features in random/inactive compounds was decisive for their correct predictions. By contrast, features absent in active and present in inactive compounds made only very little or no contributions.

Overall, for active compounds, the average sum of the SVERAD Shapley values for SVM of present features was 11.65, indicating strong positive contributions to predictions far beyond the expected value (− 4.61). On the contrary, absent features, with an average sum of Shapley values of − 1.79, made small negative contributions. RF displayed a similar behavior for active, but not for inactive compounds. Here, the average sum of the Shapley values for present and absent features was 0.51 and − 0.07, respectively, and the expected value was 0.49. Accordingly, for inactive compounds, SVM predictions were largely determined by the expected value, given that features present in these compounds slightly opposed correct predictions (with average positive contributions of 1.46) while the effect of absent features was negligible (− 0.008). By contrast, for RF, absent features made strong contributions (− 0.40 with respect to the expected value), while the average contribution of present features was only modest (0.018).

**Figure 3:**
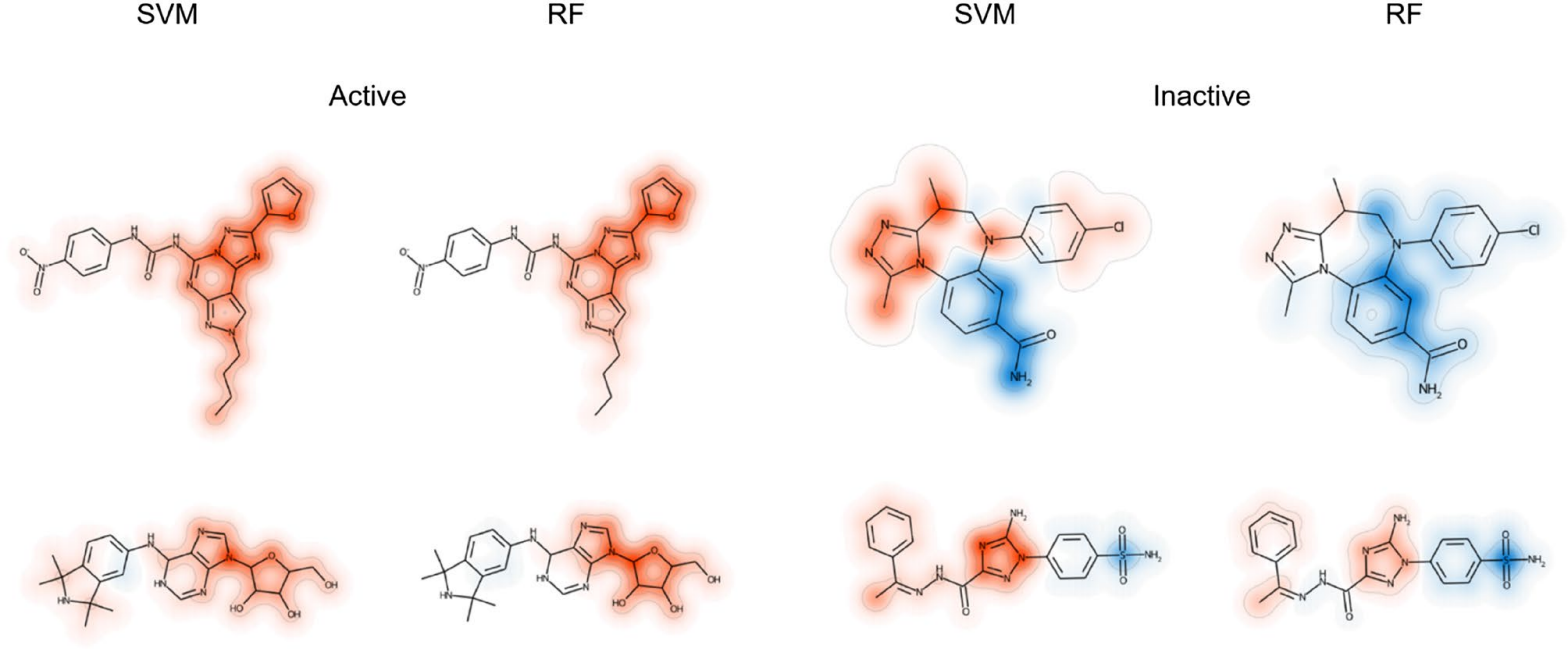
Feature mapping. Features present in exemplary active and random compounds correctly predicted by SVM and RF models are mapped on corresponding atoms. Red and blue coloring indicates positive and negative contributions towards prediction of activity and randomness, respectively.

Features with largest contributions to predictions were visualized by mapping them on the corresponding atoms in correctly predicted test compounds, as shown in Fig. 3.

For SVM and RF, features present in active compounds mostly had large positive Shapley values (red) and hence supported correct predictions (despite different value distributions, as discussed above). By contrast, for random compounds, different contributions of present features were observed. While some features supported correct predictions (blue), others opposed them (red). In active test compounds, present features supporting correct predictions with SVM and RF delineated very similar substructures.

### Computational complexity

For SVERAD, the computation of the Shapley values for a given instance has at most $O\left({\left|F\right|}^{2}\right)$ complexity, with $\left|F\right|$ being the total number of features.

We consider $U$ the number features present in the union of the explained sample with a support vector ($U=I+D$). No computation is needed for features not present in either the intersection nor the difference (${\phi }_{f}=0$). However, in the worst case, all features are present in the union, so $U=\left|F\right|$ for all the support vectors.

For each support vector, we need to compute the Shapley value for one feature from the intersection and one feature from the symmetric difference. This computation requires $O\left(1\right)$ for the intersection (hence, one only needs to calculate the inverse multinomial coefficient, as shown above), and $O\left(D\cdot \left(I+1\right)\right)=O\left(D\cdot I\right)$ for the symmetric difference. Here, $D\cdot \left(I+1\right)$ represents the size of the Cartesian product describing unique combinations of intersecting and symmetric difference features, also considering the empty coalition. Highest complexity would result from $D=\frac{\left|F\right|}{2}$ and $I=\frac{\left|F\right|}{2}$, leading to $O\left(\frac{\left|F\right|}{2}\cdot \frac{\left|F\right|}{2}\right)=O\left(\frac{|F{|}^{2}}{4}\right)=O(|F{|}^{2})$*.*

The step above must be repeated and summed up for each support vector, hence the complexity becomes $O\left({\left|F\right|}^{2}\right)\cdot N_v$. Assuming the number of support vectors $N_v$ to be a constant and given that the rest of the operations are products and sums with constant values, the final complexity will be $O(|F{|}^{2})$.

Notably, for sparse input vectors such as for the calculations reported herein, the number of features in the union $U$ was on average two orders of magnitude smaller than the total number of features $\left|F\right|$. Accordingly, in such cases, highest possible complexity is unlikely to occur. In this case, considering $U$ as the average number of features in the union between the input sample and the support vectors, the computations require on average $O\left({U}^{2}\right)$.

It follows that SVERAD has at most quadratic time requirements with respect to the number of features $\left|F\right|$ instead of exponential computation typically required for systematic Shapley value calculations.

## Conclusions

In this work, we have introduced SVERAD, a novel methodology for the computationally efficient calculation of exact Shapley values for SVM predictions with RBF kernels. The study follows and further extends a previous investigation determining exact Shapley values for the SVM/Tanimoto kernel combination, which is preferentially used for applications focusing on the assessment of chemical similarity. The SVM/RBF kernel combination (including the Gaussian kernel) is more widely applied. In the XAI field, the Shapley value concept experiences increasing interest for rationalizing predictions of ML models. Due to the complexity of explicit Shapley value calculations, approximations are typically required, for which the SHAP approach has been a pioneering development. However, low correlation between exact Shapley values calculated with SVERAD for the RBF kernel and SHAP values clearly indicated the need to use exact Shapley values for explaining SVM predictions, in marked contrast to RF. Comparative Shapley value/SHAP analysis also revealed that highly accurate SVM and RF compound predictions were determined by different relative contributions of features present or absent in active and random test compounds. However, features present in active test compounds that consistently contributed to correct predictions with both algorithms delineated corresponding substructures. Taken together, the results reported herein indicate that SVERAD substantially aids in rationalizing SVM predictions in pharmaceutical research and other scientific fields. Therefore, SVERAD is made freely available as a part of our study.

## Methods

### Compounds

For compound-based Shapley value calculations and activity predictions, we used a set of 287 A3 ligands from ChEMBL^34^ with curated high-confidence activity annotations, as reported previously.^30^ As negative data, an equally sized set of other ChEMBL compounds was randomly selected.

### Molecular representation

Compounds were represented as a keyed Morgan fingerprint with bond radius 2 (that is, a binary feature vector in which each bit position represents a unique feature)^32,35^ calculated using RDKit^36^. The fingerprint comprises compound-specific numbers of layered atom environments, which represent topological structural features^35^. Each compound is described using 5487 possible binary features.

### Machine learning models

Compounds activity predictions were carried out using SVM and RF models derived using the Scikit-learn library for Python^37^. The data set comprising active and random compounds was divided into training (50%) and test (50%) sets. The training set was then used for grid search hyperparameter optimization via cross-validation by randomly partitioning the compounds 10 times into training (50%) and validation (50%) subsets.

#### Support vector machine

Hyperparameters *gamma* (values were searched in $\left[0.0001, 0.001, 0.01, 0.1, 1, 10, 100\right]$) and *C* (values search in $[0.1, 1, 10, 50, 100, 200, 400, 500, 750, 1000]$) were optimized. Parameter *gamma* corresponds to the $\gamma$ value in the RBF kernel, as discussed above, and *C* controls the applied regularization. Smaller values of *C* favor generalization but increase the risk of training errors. Large values lead to a harder margin and strict misclassification penalties instead, thereby improving classification accuracy of training samples but potentially limiting the generalization ability. After grid search optimization, the best model with *gamma* = 0.01 and *C* = 10 produced an accuracy of 93% on the test set.

#### Random forest

The hyperparameters *n_estimators* (10, 100, 250, 500), *min_samples_split* (2, 3, 5, 7, 10), and *min_samples_leaf* (1, 2, 5, 10) were optimized. These parameters account for the number of decision trees, the minimum number of samples required to split an internal node, and minimum number of samples required to reach a leaf node, respectively. The last parameters control overfitting and model complexity. Best hyperparameter values selected via grid search were 500, 2, and 1, respectively. The final model reached an accuracy of 92% on the test set.

#### Shapley additive explanations

The Python SHAP^20^ package was used for KernelSHAP and TreeExplainer calculations. For both SVM and RF, the KernelSHAP background sample was composed of 100 randomly selected training instances. For TreeExplainer, the entire training set was used as background sample and interventional feature perturbation was used to control input feature correlation^38^.

## Data Availability

The code and data generated in this study are freely available on GitHub at: https://github.com/AndMastro/SVERAD.
